# Comparative Efficacy of Ribosome-Inactivating Protein-Containing Immunotoxins in 2D and 3D Models of Sarcoma

**DOI:** 10.3390/toxins17060308

**Published:** 2025-06-18

**Authors:** Giulia Calafato, Massimo Bortolotti, Letizia Polito, Andrea Bolognesi

**Affiliations:** 1 IRCCS Azienda Ospedaliero-Universitaria di Bologna, 40138 Bologna, Italy; giulia.calafato@aosp.bo.it; 2 Department of Medical and Surgical Sciences—DIMEC, Alma Mater Studiorum, University of Bologna, 40126 Bologna, Italy; massimo.bortolotti2@unibo.it

**Keywords:** rRNA N-glycosylases, ribosome-inactivating proteins, immunotoxins, immunoconjugates, targeted therapy, sarcoma, spheroids, organoids

## Abstract

Sarcomas are very complex and clinically challenging mesenchymal tumors. Although the standard therapeutic approach has improved the 5-year survival rate, many patients experience local relapses and/or distant metastases. To improve patient outcome, new strategies need to be investigated. Immunotoxins (ITs) based on rRNA N-glycosylases (also named ribosome-inactivating proteins, RIPs) are promising tools for cancer therapy because, by combining rRNA-glycosylase’s high cytotoxicity with carrier selectivity, they can specifically eliminate target neoplastic cells. In the last few years, 3D models have been extensively used in cancer research, particularly for target-specific drug screening. This study aimed to evaluate the possibility of utilizing ribosome-inactivating protein (RIP)-containing ITs to selectively target TfR1-, EGFR1- and Her2-expressing sarcoma adherent cells (ACs), spheroids (SSs) and organoids (ORs). To compare Its’ efficacy and ability to induce apoptosis, we performed dose–response viability and caspase 3/7 activation assays on rhabdomyosarcoma and osteosarcoma ACs, SSs and ORs treated with Tf-IT, αEGFR1-IT and αHer2-IT. Our results indicate that, compared to the corresponding unconjugated RIPs, all ITs showed increased cytotoxicity in sarcoma ACs. Despite the increased complexity characterizing 3D models, the higher IC_50_ differences between ITs and unconjugated RIPs were obtained in ORs, which appeared more resistant to the nonspecific killing of the RIPs than either the ACs or SSs, thus augmenting the therapeutic window between unconjugated and conjugated RIPs. IT induced a more delayed apoptosis in 3D compared to 2D models. Our results provide essential outcomes for the potential use of these RIP-based ITs as a therapeutic strategy to treat sarcoma.

## 1. Introduction

Sarcomas are heterogeneous tumors comprising more than 100 characterized histotypes, with a distinct age distribution, site of presentation, biologic behavior and prognosis. Among the different sarcoma subtypes, embryonic rhabdomyosarcoma, osteosarcoma and Ewing’s sarcoma represent the most widespread ones, especially in children and young adults [1]. Although the use of a multimodal approach, combining surgery, chemotherapy and radiotherapy, has improved the 5-year survival rate of affected patients, this strategy does not often prove to be conclusive, as many of these patients relapse, becoming refractory to conventional therapy and undergoing metastasis with a drop in the 5-year-survival ratio from 80% to 10–20% [2]. To improve patient outcome, new therapeutic strategies, involving the use of monoclonal antibody (mAb)-based immunotherapy, have been investigated [3]. Among them, immunotoxins (ITs) composed of mAbs linked to plant toxic enzymes, known as ribosome-inactivating proteins (RIPs) and characterized by rRNA N-glycosylases activity, can represent a valid option. Indeed, combining the selectivity of mAbs with the high cytotoxicity of RIPs, ITs have shown promising results in preclinical and clinical studies [4,5]. The use of ITs assumes that target cancer cells overexpress the antigen against which ITs are directed. Accordingly, it has been shown that the transferrin receptor 1 (TfR1) and the epidermal growth factor receptors 1 and 2 (EGFR1, Her2) are overexpressed in early and advanced sarcoma [6,7,8], thus representing potential targets of interest in IT-based sarcoma therapy. Moreover, since RIPs can kill cells through multiple death pathways, they can overcome the drug-resistance mechanisms that continue to be the principal limiting factor to achieving cure in patients with cancer [9].

In the study of novel personalized therapeutic approach, 3D-culture systems are receiving great attention because, differently from traditional 2D adherent cell cultures (AC), they recap tumor histology and the genetic and structural complexity of the original tumor, overcoming the limitations of 2D cultures and proving to be a more accurate model for in vitro cancer research [10]. To date, organoids (ORs) and spheroids represent the most promising 3D models to study the neoplastic disease [11].

Although many studies have elucidated the effectiveness of ITs in 2D cell cultures, very few data are available for IT efficacy in 3D cell cultures and even less in 3D sarcoma cultures. This study aimed to evaluate the possibility to utilize type 1 RIP-containing conjugates, hereafter referred to as ITs, to selectively target TfR1-, EGFR1- and Her2-expressing sarcoma cells in bidimensional and tridimensional models. Three target-specific ITs were tested to address their antitumor efficacy on rhabdomyosarcoma and osteosarcoma ACs and on corresponding cell-derived single spheroids (SSs) and organoids (ORs), providing useful information for potential future clinical use of these RIP-based ITs for sarcoma therapy.

## 2. Results

### 2.1. Generation of 3D Single Spheroids and Organoids from Sarcoma Cell Lines

**Figure 1:**
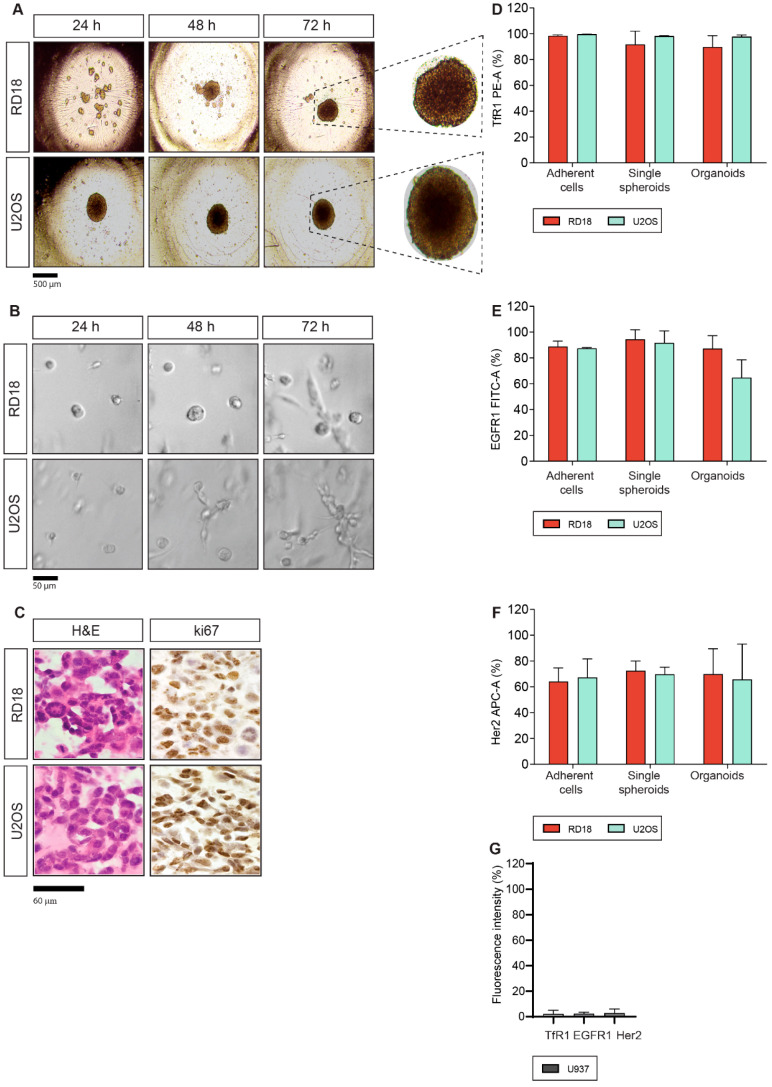
Establishment of 3D models and antigen expression. (**A**) Time-course formation assay of RD18 and U2OS single spheroids (SSs). In total, 5,000 cells/well were seeded in DMEM-F12 complete medium in an ultra-low attachment 96-well plate. Single spheroid formation was monitored daily, using a phase contrast microscope with a digital camera (40× magnification); scale bar = 500 µm (beside, a further 3× zoom of 3D spheroids is shown). (**B**) Time-course formation assay of RD18 and U2OS maxi-ring organoids (ORs). In total, 20,000 cells/well were seeded in 24-well plates in a cold Mammocult–Matrigel mixture. OR formation was monitored daily, using Celigo S Software (bright-field mode); scale bar = 50 µm. (**C**) H&E and ki67 staining of formalin-fixed paraffin-embedded slides of RD18- and U2OS-derived ORs. Images were acquired through a microscope system (400× magnification); scale bar = 60 µm. (**D**) Flow cytometry analysis of TfR1 expression in RD18 and U2OS ACs, SSs and ORs. *Y* axis reports the mean of the antiTfR1-PE intensity (%). (**E**) Flow cytometry analysis of EGFR1 expression in RD18 and U2OS ACs, SSs and ORs. *Y* axis reports the mean of antiEGFR1-FITC intensity (%). (**F**) Flow cytometry analysis of Her2 expression inRD18 and U2OS ACs, SSs and ORs. *Y* axis reports the mean of the antiHer2-APC intensity (%). (**G**) Flow cytometry analysis of TfR1, EGFR1 and Her2 expression in U937 ACs. *Y* axis reports the mean of the fluorescence intensity (%). Experiments were independently repeated two (flow cytometry) or three (time-course formation assays) times, each conducted in triplicate, and values are reported as mean ± SD. Data were analyzed with a one-way ANOVA followed by Tukey’s post-hoc test (Prism v8).

To assess and compare the antitumor effects of the selected ITs on rhabdomyosarcoma (RD18) and osteosarcoma (U2OS) adherent cells (AC) and cell-derived 3D models, we generated single spheroid (SS) and organoid (OR) cultures. Time-course assays were performed to optimize the appropriate formation times and culture conditions for both SS and OR cultures. As shown in Figure 1A, RD18 and U2OS SSs were generated in ultra-low attachment (ULA) plates in DMEM-F12 complete medium within a culture time of 72 h and 24 h, respectively. This procedure allowed the formation of one SS for each well of the ULA plate with a homogeneous size (about 500 µm), compact morphology and a well-circumscribed edge. RD18- and U2OS-derived ORs were generated as described in Section 5.1. The generation of ORs with appropriate density was achieved within 48 h (Figure 1B). The morphological characterization of the OR was assessed through hematoxylin and eosin (H&E) staining, while their proliferative activity was checked through Ki67 immunohistochemistry staining (Figure 1C). RD18 and U2OS ORs presented a lower size compared to SSs and were characterized by a peculiar shape of small clusters grouped in a ring with a strong positivity for the tumor proliferation marker ki67 (Figure 1C).

### 2.2. TfR1, EGFR1 and Her2 Antigen Expression

The analysis of antigen expression is a fundamental preliminary step to investigate the targeted antitumor effects of ITs. Accordingly, TfR1, EGFR1 and Her2 expression levels were analyzed by flow cytometry in RD18 and U2OS ACs, SSs and ORs. Interestingly, RD18 and U2OS ACs showed high TfR1 expression, which was maintained also in RD18- and U2OS-derived SSs and ORs within similar intensity ranges (Figure 1D). Similarly, EGFR1 maintained a high level of expression in 2D and 3D models, even if the antigen expression slightly decreased in U2OS ORs (Figure 1E). Her2 antigen expression was in the same way high in all models, albeit lower than those reported for TfR1 and EGFR1 (Figure 1F). These data highlighted that the increased complexity characterizing 3D models did not impair the intensity of antigen expression. To verify whether TfR1, EGFR1 and Her2 were selectively expressed on target cells, we decided to also evaluate their expression on the monocyte-derived cell line U937 AC through flow cytometry. Results indicated that in U937 ACs expressed all the selected antigens only at very low levels (≤2.8%) (Figure 1G). Flow cytometry results indicated that, compared to RD18/U2OS ACs, SSs and ORs, U937 ACs showed a statistically significant decrease in TfR1 (*p* < 0.0001, compared to RD18/U2OS models), EGFR1 (*p* = 0.0041, *p* = 0.0028, *p* = 0.0166 compared to RD18 ACs, SSs and ORs, respectively; *p* = 0.0045, *p* = 0.0034, *p* = 0.023 compared to U2OS ACs, SSs and ORs, respectively) and Her2 expression (*p* = 0.0422, *p* = 0.0225, *p* = 0.0274 compared to RD18 ACs, SSs and ORs, respectively; *p* = 0.0331, *p* = 0.0274, *p* = 0.0373 U2OS ACs, SSs and ORs, respectively) (Table 1, Figure 1E–G; Figures S1–S4). U937 cells were not able to grow either as SSs or as ORs. Indeed, U937 viability progressively decreased when cells were cultured as SSs in DMEM-F12 complete medium in ULA plates (Figure S5A) or as ORs (cell–Matrigel mixture) in Mammocult complete medium (Figure S5B). Therefore, we performed the analysis only on U937 ACs. Accordingly, U937 cells were used as a non-target cell line.

An overview of the target expression analysis in 2D and 3D sarcoma models is reported in Table 1, while detailed flow cytometry plots of the selected antigens, across all experimental models, are shown in Figures S1–S4.

### 2.3. Selective Efficacy of RIP-Containing ITs in RD18- and U2OS-Derived Models

**Figure 2:**
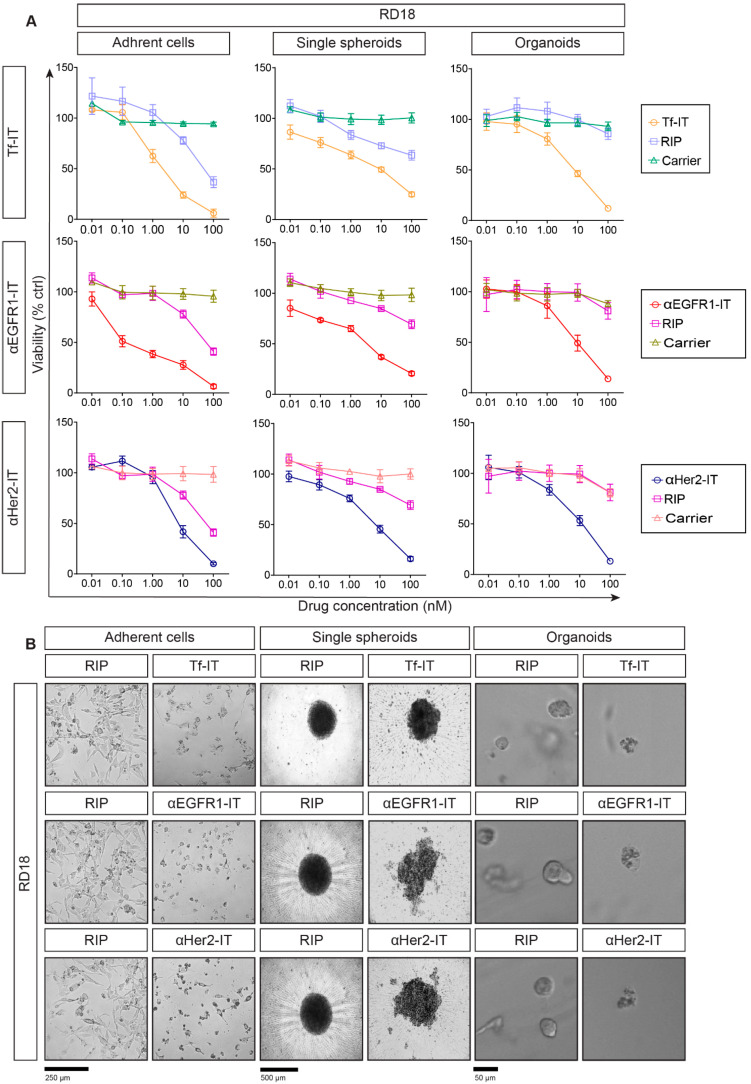
Selective efficacy of RIP-containing ITs in RD18-derived 2D and 3D models. (**A**) Dose–response curves in RD18 adherent cells (ACs), single spheroids (SSs) and organoids (ORs) treated for 72 h at the indicated concentrations of ITs, unconjugated RIPs or carriers. Viability was evaluated by MTS reduction (ACs) or ATP production (SSs and ORs). Experiments were independently repeated three (ACs and ORs) or four times (SSs), each in triplicate. (**B**) Representative images of RD18 ACs, SSs and ORs treated with the indicated ITs or RIPs for 72 h at 100 nM. AC and SS images were obtained using a phase-contrast microscope equipped with a Nikon Eclipse TS100 camera, while OR images were obtained with the Celigo S Imaging Cell Cytometer (bright-field mode). IT, RIP or carrier viability values are expressed as a percentage of controls (PBS) and reported as mean ± SD (Prism v8).

**Figure 3:**
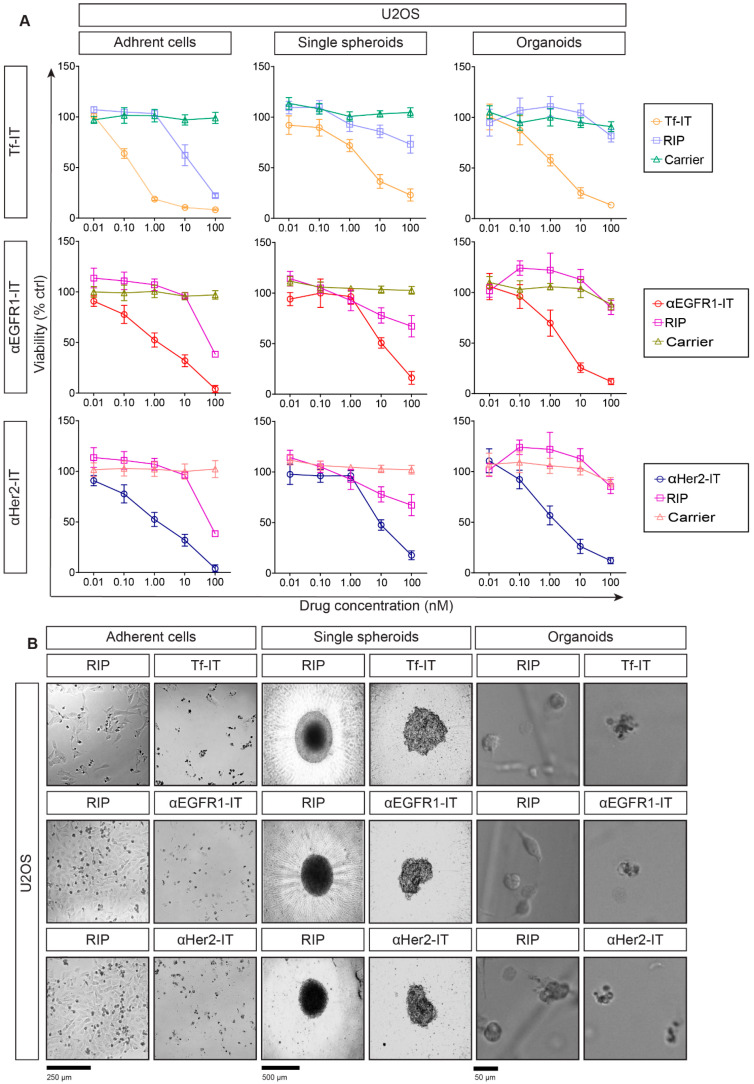
Selective efficacy of RIP-containing ITs in U2OS-derived 2D and 3D models. (**A**) Dose–response curves in U2OS adherent cells (ACs), single spheroids (SSs) and organoids (ORs) treated for 72 h with the indicated concentrations of ITs, unconjugated RIPs or carriers. Viability was evaluated by MTS reduction (ACs) or ATP production (SSs and ORs). Experiments were independently repeated three (ACs and ORs) or four times (SSs), each in triplicate. (**B**) Representative images of U2OS ACs, SSs and ORs treated with the indicated ITs or RIPs for 72 h at 100 nM. AC and SS images were obtained using a phase-contrast microscope equipped with a Nikon Eclipse TS100 camera, while OR images were obtained with the Celigo S Imaging Cell Cytometer (bright-field mode). IT, RIP or carrier viability values were expressed as a percentage of controls (PBS) and reported as mean ± SD (Prism v8).

Based on flow cytometry results, we assessed the selective antitumor effect of the three ITs, Tf-IT, αEGFR1-IT and αHer2-IT, in 2D and 2D sarcoma models. ACs, SSs and ORs were treated for 72 h with scalar concentrations of the tested substance, ranging from 0.01 nM to 100 nM for ITs or carriers and from 0.01 nM to 10,000 nM for unconjugated RIPs. To facilitate the comprehension of the data, we show IT, RIP and carrier concentration ranges from 0.01 nM to 100 nM in the main figures (Figure 2A,B), whereas the complete RIP viability curves (from 0.01 nM to 10,000 nM) are shown in Figure S6. IT efficacy was assessed through MTS- (ACs) or 3D ATP-based viability assays (SSs and ORs) in dose–response curves (Figure 2A, Figure 3A and Figure S6). Representative images of RD18 and U2OS treated for 72 h at 100 nM with ITs or RIPs are reported in Figure 2B and Figure 3B. IT and RIP concentrations inhibiting cell viability by 50% (IC_50_) are reported in Table 2. All ITs were much more cytotoxic toward target-positive ACs compared to unconjugated free RIPs or carriers (Figure 2A and Figure 3A). Although the 3D models used were more complex and difficult to penetrate than 2D ones, ITs were more effective in 3D models. In fact, the wider IC_50_ differences between ITs and unconjugated RIPs were obtained in SSs and ORs (Figure 2A, Figure 3A and Figure S6, Table 2). This might be due to the different specific (receptor-mediated) or nonspecific (pinocytosis) entry of RIPs in 3D versus 2D models.

To support our data, representative imaging of viability assay showed that at the highest concentration (100 nM), RIP-containing ITs almost completely eliminated RD18 and U2OS ACs, SSs and ORs, while RIPs had a lower cytotoxic effect (Figure 2B and Figure 3B), thus highlighting the higher efficacy of ITs over RIPs alone. To confirm the target-specific cytotoxicity of ITs, we tested ITs on the non-target cell line U937, characterized by a very low expression of TfR1, EGFR1 and Her2 (Figure 1G and Figure S4). Since U937 cells were not able to grow as SSs or ORs (Figure S5A,B), we analyzed the IT, RIP and carrier cytotoxicity on the U937 ACs through an MTS assay. Viability dose–response curves indicated that even at the highest tested dose (100 nM), the ITs killed only 20–40% of cells showing IC_50_s > 100 nM (Figure S5C and Table 2), without any relevant change in U937 cell morphology (Figure S5D).

In RD18, ITs showed a higher targeted efficacy compared to RIPs or carriers alone both in 2D and 3D models (Figure 2A and Figure S6). The Tf-IT IC_50_ values were 1.3, 1.8 and 1.9 logs lower than the unconjugated RIP ones in ACs, SSs and ORs, respectively (Table 2). The αEGFR1-IT IC_50_ values were 2.1–2.4 logs lower than that of unconjugated RIPs in each model (Table 2). The αHer2 ITs showed a similar behavior, the related IC_50_ values being 1.3 (ACs), 2.0 (SSs) and 1.6 (ORs) logs lower compared to the corresponding RIP IC_50_ values (Table 2). Interestingly, all the unconjugated carriers (Tf, αEGFR1 and αHer2), showed no or a very low reduction in viability only at the higher tested concentration, with IC_50_ values >100 nM (Figure 2A, Table 2). To further support the above reported results, representative images of RD18 ACs, SSs and ORs treated with 100 nM ITs or the corresponding RIPs for 72 h were generated. These experiments showed that the treatment with ITs determined consistent changes in the morphology both in 2D and 3D cell models, inducing cell shrinkage and rounding in ACs and a disruption of the compact morphology in SSs and ORs (Figure 2B). At the same concentration, unconjugated RIPs were unable to induce any of the cell changes described for ITs (Figure 2B).

As for RD18, also in U2OS models, the ITs showed a higher selective cytotoxicity with respect to unconjugated RIPs or carriers (Figure 3A and Figure S6). Specifically, Tf-IT showed IC_50_ values 2.0–2.4 logs lower than those of free RIPs (Table 2); the αEGFR1-IT had IC_50_ values 1.70–2.0 logs lower than unconjugated RIP (Table 2); the αHer2-IT showed IC_50_ values 1.3–2.5 logs lower than unconjugated RIP (Table 2). Interestingly, in U2OS ORs, all ITs decreased organoids viability by about 50–60% at 1 nM. At the same concentration, RIPs were not able to induce any cytotoxic effect. As for RD18, all the unconjugated carriers had IC_50_ values >100 nM. Moreover, as to that observed in RD18, ITs provoked cell shrinkage and rounding in U2OS ACs and the disappearance of the compact morphology and demarcated edges in SSs and ORs (Figure 3B). To note, both in RD18 and U2OS, unconjugated RIPs were less effective in 3D models than in 2D, possibly due to substantial differences in architectural configuration and nutrient or drug diffusion along ACs, SSs and ORs. Nevertheless, the therapeutic window (difference in activity between the IC_50_ of unconjugated and carrier-conjugated RIPs) was always wider in the 3D models, even though there was some variability between the two cell lines.

### 2.4. Evaluation of Cell Death Mechanisms Induced by the ITs in RD18 and U2OS 2D and 3D Models

**Figure 4:**
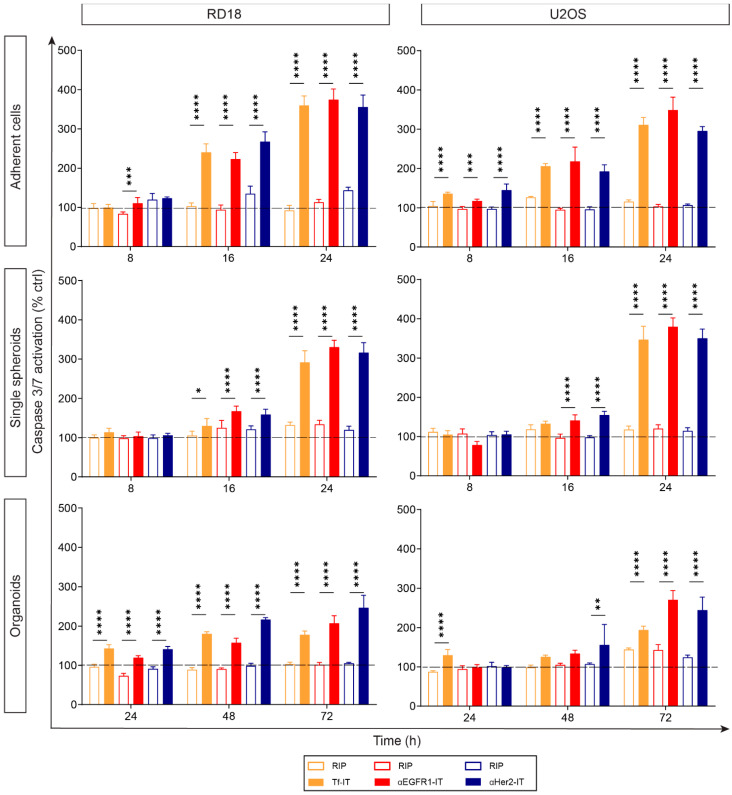
Time-dependent caspase 3/7 activation in RD18 and U2OS 2D and 3D models. Caspase 3/7 activation in RD18 and U2OS adherent cells (ACs), single spheroids (SSs) and organoids (ORs) exposed to IC_50_ of ITs or corresponding RIPs. Caspase 3/7 activation was assessed with the Caspase-Glo 3/7 assay system (ACs) and Caspase-Glo 3/7 3D assay system (SSs and ORs). Experiments were independently repeated two (ACs and ORs) or four (SSs) times, each in triplicate. For each condition, the caspase 3/7 activation value was normalized to the corresponding viability value and expressed as a percentage of untreated controls (PBS). The data were reported as mean ± SD and analyzed with a one-way ANOVA followed by Tukey’s post-hoc test. * *p* < 0.05, ** *p*  <  0.01, *** *p*  <  0.001, **** *p* < 0.0001 (Prism v8).

We decided to evaluate whether ITs could induce apoptosis not only in RD18 and U2OS AC cultures but also in corresponding SS and OR cultures and to compare the timing of apoptosis activation in the different models. For this aim, we tested the activation of the effector caspase 3/7, in RD18 and U2OS ACs, SSs and ORs either treated with each IT (at IC_50_ concentration) or with the corresponding RIP used at the same concentration of the IT (Figure 4). Caspases 3/7 activation was measured after 8, 16 and 24 h of treatment in all models; considering the matrix-like related difficulty of the molecule diffusion characterizing organoids, two additional time points (48 and 72 h) were added in this model. To correlate caspase activation with cytotoxicity, parallel cell viability assays were performed in the same experimental conditions (Figure S7). Results showed that, compared to the corresponding unconjugated RIP, Tf-IT, αEGFR1-IT and αHer2-IT doubled and tripled caspase 3/7 activation at 16 h and 24 h, respectively, in RD18 and U2OS ACs (*p* < 0.0001) (Figure 4). Overall, caspase 3/7 activation profile was similar among the ITs tested and between RD18 and U2OS ACs (Figure 4). The results obtained in SS showed that the activation of caspase 3/7 was delayed compared to what was observed in ACs (Figure 4). In SSs, we observed that at 16 h all ITs induced a lower caspase activation value than in ACs, albeit statistically significant compared to that induced by RIPs (*p* < 0.0001). Nevertheless, at 24 h, all ITs induced a caspase 3/7 activation value in SSs almost identical to that induced in ACs (Figure 4). Surprisingly in ORs, differently from ACs and SSs, we did not detect IT-induced caspase 3/7 activation at 8 and 16 h, and it was very poor or not present at 24 h (Figure S7C,D). According to this, and considering the delayed apoptosis activation characterizing SS cultures, we decided to add two additional time points at 48 h and 72 h (Figure 4 and Figure S7C,D). We found that in RD18 ORs, all ITs doubled the caspase 3/7 activation as compared to RIPs at 48 h and 72 h (*p* < 0.0001), while in U2OS ORs, we observed this trend at 72 h (*p* < 0.0001) (Figure 4). Our results indicated that, although ITs promoted the strong activation of caspase 3/7 in both 2D and in 3D sarcoma models, SSs and ORs presented a delayed activation time, compared to ACs (delay of 8 h and 32–56 h, respectively).

## 3. Discussion

Although sarcomas are relatively rare, they are extremely widespread in children and young adults. The standard treatment regimen does not often prove to be conclusive, as many patients with localized primary sarcoma can experience relapses, progression and metastasis [12]. Immunotherapy is one of the most promising therapeutic approaches for the treatment of cancer. Numerous clinical trials have reported the effects of antibody-based therapies on a variety of tumors, including sarcoma, in terms of improving overall survival compared to conventional chemotherapy drugs [13]. Antibodies (or other carriers) linked to rRNA N-glycosylases (also named ribosome-inactivating proteins, RIPs) are promising tools for cancer therapy because they combine rRNA N-glycosylase’s high cytotoxicity with mAb’s selectivity [4]. To evaluate novel personalized treatments for cancer patients, tridimensional models, such as spheroids and organoids, are the gold standard for disease modeling and drug screening and development, as they better resemble the features of the original tumor [14].

Based on these considerations, this study aimed at assessing the selective antitumor efficacy of three RIP-containing ITs. These ITs, namely Tf-IT, αEGFR1-IT and αHer2-IT were compared to free RIPs or carriers in 2D and 3D growing sarcoma cell line models. With respect to 2D models, 3D models, represented by spheroids and organoids, have more complex cellular interactions and display a microenvironment much more similar to the tumor one. To perform our analysis, we chose two cell lines representative of the two main types of sarcoma subtypes, embryonal rhabdomyosarcoma cell line RD18 and osteosarcoma cell line U2OS. ITs were selected basing on three considerations: (i) TfR1, EGFR1 and Her2 antigens have been well exploited as targets for cancer immunotherapy in several carcinoma models [15,16,17,18]; (ii) the overexpression of these antigens was reported in primary or advanced sarcoma [6,7,8]; (iii) ITs directed against those antigens were previously evaluated in some carcinoma and sarcoma cell lines, showing high cytotoxicity [19,20,21]. Single spheroids and organoids can be used for different but also overlapping scientific purposes and they can be distinguished based on tumor cell sources, protocol for culture and the time required for establishment [11]. For research purposes, spheroids are mostly used to study metabolic and proliferation indexes or drug resistance mechanisms. Organoids, better resembling the histological heterogeneity and the genetic features of the original tumor, are mostly used to predict with good reliability antineoplastic drugs’ responses [22,23,24]. To assess whether the selected antigens were present in RD18- and U2OS-derived models, we analyzed TfR1, EGFR1 and Her2 expression values by flow cytometry in adherent cells (ACs), single spheroids (SSs) and organoids (ORs). Results indicated that TfR1 and EGFR1 were highly expressed in RD18 (98.9% and 91.8%, respectively) and U2OS (99.7% and 87.9%, respectively) ACs, whereas Her2 showed a slightly lower expression value (64.1% in RD18 and 67.3% in U2OS) than the other two antigens. The data obtained in the cytofluorimetric analysis of antigen expression in RD18 and U2OS ACs agreed with those reported in the literature and indicated that these surface antigens were good neoplastic targets for selective immunotherapy [19,25,26,27]. These data were strengthened by those obtained in 3D models, in which the antigen expression patterns were similar to those of the original cell lines. According to these results, we selected three ITs directed against TfR1, EGFR1 and Her2. The selected conjugates had already shown an antitumor effect in in vivo models of carcinoma [16] and in glioblastoma cell lines [15]. Moreover, EGFR-specific ITs were found to be effective in rhabdomyosarcoma cells [28,29,30]. Accordingly, our results indicate that the conjugation to the carriers enhanced the cytotoxic effect of RIPs against target cells. Indeed, in RD18 and U2OS ACs, ITs exhibited IC_50_ values 1.3–2.3 logs lower than the corresponding unconjugated RIPs. Interestingly, our results show that IT efficacy was also maintained within similar toxic ranges in the more complex and heterogeneous SS and OR 3D models. To note, the efficacy of our conjugates in 2D and 3D models were as good as those reported in the literature for other types of immunoconjugates based on a truncated form of Pseudomonas exotoxin (PE38) [31] or based on trastuzumab emtansine (T-DM1) [32,33]. On the other hand, some studies reported a decrease in immunotoxin efficacy in different types of 3D models compared to ACs, meaning that the chemical structure and intra-tumor diffusion of ITs, as well as the type of tumor and the molecular target, could be additional factors influencing the IT response in 3D models [34,35]. Our results indicate that ITs were also effectively cytotoxic in 3D models, thus confirming the antineoplastic potential of ITs in these types of tumors, even if, at the maximum IT tested dose, a residual viability of about 10–20% was present in SSs and ORs but not in ACs. Interestingly, we noted that, compared to ACs or SSs, organoids were more resistant to the nonspecific cytotoxic activity of unconjugated RIPs. This could be due to the presence of the Matrigel-based extracellular matrix (ECM), which can limit the nonspecific toxicity of RIPs. Our data show that the treatment with the unconjugated transferrin or mAbs never induced a decrease in viability in target ACs, SSs and ORs. It needs to be highlighted that the cytotoxic activity of our ITs was entirely due to RIPs, since in the experimental systems used, there were no cells of the immune system nor complement factors, which could induce ADCC or CDC, respectively [36]. It is well known that, besides the ability to resemble the morphological and molecular features of the parental tumor, three-dimensional models, especially ORs, are widely used in translational medicine to predict patient response to cancer therapy and to perform high-throughput screenings. This strengthened our results, especially considering the increased therapeutic window between unconjugated and conjugated RIPs observed in the 3D models.

The use of RIPs as payloads of conjugates has some advantages and some disadvantages over drugs. Conventional antiblastic drugs are small molecules that act in stoichiometric ratios and mainly on dividing cells. RIPs are enzymes of about 30 kDa that act on various molecular intracellular targets and are able to induce multiple cell death pathways. Due to these characteristics, RIPs are powerful cytotoxic agents, which can act even on tumors with a low replication index and on neoplastic clones presenting a blocked or impaired death pathway [37,38,39]. Moreover, the selection of clones resistant to RIPs have never been reported. The main side effect evidenced in clinical trials with RIP-based conjugates is immunogenicity [40]. However, the wide availability of RIPs belonging to unrelated plants could be useful to bypass the immunogenicity problem by the sequential administration of conjugates containing different non-cross-reacting RIPs.

The ability of RIP-containing ITs to induce mainly apoptosis is long-established evidence [41]. Considering this, we investigated whether the selected ITs could induce apoptosis under different cell growth conditions, ultimately comparing the timing and intensity of apoptosis activation in 2D and 3D models. The results of the caspase 3/7 assays showed that Tf-IT, αEGFR1-IT and αHer2-IT were able to induce caspase activation in RD18 and U2OS ACs, SSs and ORs, with a similar intensity of activation but different timing. A significant increase in IT-triggered caspase 3/7 activation was observed, both compared to controls and to unconjugated RIPs, at 16 h in ACs, at 24 h in SSs and at 48–72 h in ORs with a corresponding decrease in viability. The delayed apoptosis activation observed in SSs and ORs could be possibly linked to the difficulties encountered by ITs to penetrate the spheroid mass and Matrigel-based matrix, respectively.

## 4. Conclusions

This work is the first attempt to establish a comparative profile of RIP-containing ITs’ cytotoxic efficacy on sarcoma-derived ACs, SSs and ORs. Our results revealed that the tested ITs were efficient in both three-dimensional SS and OR models, despite the increasing structural complexity characterizing both single spheroids and organoids. Organoids were more resistant than ACs and SSs to the nonspecific cytotoxic activity of unconjugated RIPs, but they were affected by the ITs in the same manner of ACs and SSs, thus augmenting the therapeutic window between unconjugated and conjugated RIPs. Moreover, in 3D models, apoptosis was induced with the same intensity, but with a delayed timing, with respect to the 2D model. These results provide interesting insights into the study of alternative target experimental options to treat sarcoma. Further in vivo biodistribution, pharmacokinetic and systemic toxicity studies will be required prior to using these ITs in clinical trials, especially in view of the target antigens’ wide distribution pattern and RIP-related immunogenic events.

## 5. Materials and Methods

### 5.1. Culture of Adherent Cell Lines, Single Spheroids and Organoids

Human embryonal rhabdomyosarcoma RD18, human osteosarcoma U2OS and hu-man monocyte U937 cell lines were kindly provided by the Department of Medical and Surgical Sciences (University of Bologna) and validated through the GenePrint 24 System (Promega, Madison, WI, USA). RD18 ACs were cultured in DMEM (Corning, New York, NY, USA), and U2OS and U937 ACs were cultured in RPMI 1640 medium (Corning), both containing 10% heat-inactivated fetal bovine serum (FBS) (Corning), 2 mM L-glutamine (Gibco, Waltham, MA, USA), 100 U/mL penicillin and 100 µg/mL streptomycin (Sigma-Aldrich, Burlington, MA, USA). These culture media were named DMEM or RPMI complete medium, respectively. Cells were periodically checked for mycoplasma contamination. Single spheroids (SSs) from RD18, U2OS and U937 cells were obtained by seeding 5000 cells/well in ultra-low attachment 96-well U-bottom plates (ULA) (Corning) in serum-free DMEM F-12 (ThermoFisher Scientific, Waltham, MA, USA) complete medium, containing 5 µg/mL bovine insulin (Merck, Burlington, MA, USA), 20 ng/mL rEGF (Merck), 20 ng/mL bFGF (Gibco, Waltham, MA, USA), 1× B27 (Gibco), 0.5 µg/mL hydrocortisone (Merck) and 1% penicillin/streptomycin. RD18-, U2OS- and U937-derived organoids (ORs) were cultured in serum-free Mammocult complete medium (StemCell Technologies, Vancouver, Canada) containing Mammocult supplements (1:10). RD18, U2OS and U937 ORs were established as described in [42]. Briefly, cells were collected and filtered through a 100 μm strainer. After centrifuging, cells were resuspended in a 1:1.33 Mammocult–Matrigel (BD Biosciences, Franklin Lakes, NJ, USA) mixture and seeded in Mammocult complete medium according to the “ring-method”. In the 96-well plate, rings were named “mini-rings”, while in the 24-well plate “maxi-rings”.

### 5.2. RIPs, Carriers and ITs

The type I RIP saporin (SO6) was purified from *Saponaria officinalis* L. seeds while type I RIP ocymoidine (Ocy) was purified from *Saponaria ocymoides* L. seeds as described in [43,44]. To note, after the purification procedure, we obtained highly pure (>98%) RIPs, which were used for the chemical conjugation with carriers. Murine monoclonal antibody (mAb) anti-EGFR1 (Mint5) clone 528 and anti-Her2 (MGR-3) were kindly provided by Oncogene Science (Mid-Cambridge, MA, USA) and by Menarini (Florence, Italy), respectively. Transferrin-saporin (Tf-IT) is composed of the carrier transferrin (Tf) chemically linked to SO6 and was produced as reported in [45]. Although Tf-IT is not a proper IT, since the RIP is not linked to an mAb but to transferrin (Tf), we decided to denominate it Tf-IT to facilitate the comprehension of the results. Anti-EGFR1-ocymoidine (αEGFR1-IT) and anti-Her2-ocymoidine (αHer2-IT), composed of mAb αEGFR1 and αHer2, respectively, were chemically linked to Ocy following the method described in [46].

### 5.3. H&E and ki67 Immunohistochemistry

For the histology characterization of RD18 and U2OS ORs, 20,000 cells/well were seeded in 24-well plates allowing for the formation of maxi-rings, as previously described. ORs were fixed overnight in 10% buffered formalin (VWR, Radnor, PA, USA), collected, and centrifuged at 2000× *g* for 10 min at 4 °C. The cell pellet was mixed with HistoGel (ThermoFisher Scientific), and the gelled mixture was transferred to a histology cassette for standard paraffin embedding and sectioning. H&E staining was performed using Vector Labs Kit (Vector Laboratoires, Newark, CA, USA), following the manufacturer’s instruction. For immunohistochemistry staining, endogenous peroxidases were blocked with Peroxidaze 1 (Biocare Medical, Pacheco, CA, USA) at room temperature for 4 min, while heat-induced antigen retrieval was performed in an NxGENDeloaking Chamber using the Diva Decloaker solution (Biocare Medical). Slides were incubated 2 h at room temperature with anti-ki67/caspase 3 antibodies (Biocare Medical). Secondary antibody staining was performed for 30 min at room temperature with MACH 2 double Stain 2 (Biocare Medical). Only ki67 chromogen was developed with the Betazoid DAB kit (Biocare Medical). Nuclei were counterstained with Haematoxylin-1 (Vector Laboratoires). The slides were mounted with Permount (ThermoFisher Scientific). Images were acquired with a Revolve Upright and Inverted Microscope System (Echo Laboratories, San Francisco, CA, USA).

### 5.4. Flow Cytometry Analysis

For the flow cytometry staining, primary antibodies were used as follows: anti-transferrin receptor PE-conjugated mAb 1:50 (Abcam, Cambridge, UK), anti-EGFR FITC-conjugated mAb 1:10 (Abcam) and anti-ERBb2/Her2 APC-conjugated mAb 1:10 (Novus Biologicals, Centennial, CO, USA). Negative controls were represented by unstained ACs, SSs or ORs incubated with PBS-containing 1% FBS. Samples were incubated in the dark for 30 min on ice with the specific primary antibody solution (stained samples) or with PBS-containing 1% FBS (negative controls). For AC cells, 500,000 cells/sample were stained with the specific primary antibody. After 30 min, samples were washed twice, resuspended in cold PBS containing 1% FBS and analyzed through an Attune NxTFlow Cytometer (Thermo Fisher Scientific). For SSs, 5000 cells/well were seeded in ULA plates, as previously described. After SS formation, samples were incubated with trypsin/EDTA for 10 min at 37 °C to allow SS dissociation. Reaction was stopped with DMEM F12 complete medium and after centrifuging, samples were incubated with the specific primary antibody. Samples were resuspended in PBS containing 1% FBS and analyzed using a CytoflexFlow Cytometer (Beckman Coulter, Brea, CA, USA). For RD18 and U2OS-derived ORs, 20,000 cells/well were seeded in 24-well plates allowing for maxi-ring OR formation. After this, ORs were incubated for 20 min at 37 °C with the Dispase solution (Gibco) (5 mg/mL) to allow their release from Matrigel. ORs were transferred to a falcon tube, centrifuged at 800× *g* for 10 min at 4 °C and stained as described for AC.

### 5.5. Viability Assays

For 2D cultures, 5000 cells/well were seeded in 96-well plates (Corning) in DMEM complete medium (RD18) or RPMI complete medium (U2OS and U937). After 24 h, ACs were treated for 72 h with scalar dilutions of RIPs, ITs or carriers in DMEM (RD18) or RPMI (U2OS and U937) complete media. AC viability was measured using the MTS-based colorimetric CellTiter 96^®^ AQueous One Solution Cell Proliferation Assay (Promega), following the manufacturer’s instructions. Absorbance at 492 nm was measured by the microplate reader Multiscan EX (ThermoFisher Scientific). For SSs, 5000 cells/well were seeded in ULA plates in DMEM-F12 complete medium. SSs were treated for 72 h with scalar dilutions of RIPs, ITs or carriers in DMEM F-12-complete medium, and viability was analyzed using the ATP-based luminometric CellTiter-Glo^®^ 3D Cell Viability kit (Promega), following the manufacturer’s instructions. The luminescent signal was measured by a Fluoroskan Ascent FL (Lab-Systems Diagnostics, Vantaa, Finland) (integration time 1 s). For cell-derived ORs (1000 cells/well), cells were seeded according to the “mini-ring” method in 96-well plate (Corning) in Mammocult complete medium. After 48 h, cell-derived ORs were treated for 72 h with scalar dilutions of RIPs, ITs or carriers in Mammocult complete medium. After treatment removal, cells were incubated at 37 °C for 25 min in a Dispase solution (5 mg/mL). Plates were shaken for 5 min at 80 rpm. The ATP-based luminometric CellTiter-Glo 3D assay (Promega) was used to measure organoids’ viability according to the company’s instructions. The luminescent signal was measured by a SpectraMax iD3 (Molecular Devices, San Jose, CA, USA) (integration time 500 ms). For the AC, SS and OR treatments, scalar concentrations ranging from 0.01 nM to 10,000 nM (RIP) and from 0.01 nM to 100 nM (IT and carrier) were used. To facilitate the comprehension of the data, we show IT, RIP and carrier concentration ranges from 0.01 nM to 100 nM in the main figures (Figure 2A,B), whereas the complete RIP viability curves (from 0.01 nM to 10,000 nM) are shown in Supplementary Materials, Figure S6. For each condition, viability was normalized on the untreated controls (PBS, vehicle) and expressed as a percentage of mean value ± standard deviation (SD).

### 5.6. Imaging

The morphological analysis of ACs and SSs in 96-well plates was carried out using a phase-contrast microscope with a digital camera (Nikon, Tokyo, Japan). OR imaging was assessed through a Celigo S Imaging Cell Cytometer (Nexcelom) in bright-field mode, using the best two focal planes of confluence in order to capture the maximum number of ORs per focal plane.

### 5.7. Caspase 3/7 Activation Assays

Caspase 3/7 activation was evaluated through the Caspase-Glo^®^ 3/7assay system (Promega) for ACs and Caspase-Glo^®^ 3/7 3D assay system (Promega) for SSs and ORs, following the manufacturer’s instructions. Treatments were carried out with the concentration inhibiting 50% of the cell viability (IC_50_) of the selected IT, obtained from the relevant 72 h dose–response curve, and the same concentration was chosen for the unconjugated RIP. RD18 or U2OS ACs, SSs and ORs were seeded as described for the viability experiments. In ACs and SSs, treatments were carried out for 8, 16 or 24 h in ACs and SSs, whereas in ORs, two extra time points were added (48 h and 72 h). The luminescent signal was measured with a Fluoroskan Ascent FL (integration time 10 s) for ACs and SSs and with a SpectraMax iD3 (integration time 500 ms) for ORs. For each condition, the caspase 3/7 activation value was first normalized to the corresponding viability value and then expressed as the percentage of untreated controls (PBS, vehicle).

### 5.8. Statistical Analysis

The statistical analysis was performed with Prism v8 software (Graph Pad Software Inc., La Jolla, California, USA). The normal distribution of the data was confirmed by the Shapiro–Wilk test. A one-way ANOVA with Tukey’s post-hoc test was used for multiple comparisons of the mean differences among groups. Statistical significance was assessed as * *p* < 0.05, ** *p* < 0.01, *** *p* < 0.001, **** *p* < 0.0001. IC_50_ was calculated using non-linear regression (Prism v8).

## Figures and Tables

**Table 1:** Overview of cytofluorimetric analysis of antigen expression in 2D and 3D sarcoma models. Expression of TfR1, EGFR1 and Her2, in RD18 and U2OS adherent cells (ACs), single spheroids (SSs) and organoids (ORs) and in U937 negative control cells. Values are expressed as the mean of the FITC, APC or PE intensity (%) evaluated by flow cytometry. Data represent the mean of two independent experiments, each performed in triplicate. SD never exceeded 6%.

	Antigen Expression (%)						
	RD18			U2OS			Non-Target U937
	AC	SS	OR	AC	SS	OR	AC
TfR1	98.5	91.8	89.8	99.7	98.3	97.7	2.1
EGFR1	88.9	94.4	87.3	87.5	91.6	64.8	2.3
Her2	64.1	77.4	69.9	67.3	69.8	65.7	2.8

**Table 2:** Comparison of IC_50_ values calculated from dose–response curves in 2D and 3D sarcoma models. RD18 and U2OS adherent cells (ACs), single spheroids (SSs) and organoids (ORs) and U937 non-target cells were treated with ITs or unconjugated RIPs for 72 h. IC50s were calculated using a non-linear regression of viability data from Figure 2, Figure 3 and Figure S6 (Prism v8).

	IC_50_ (nM)						
	RD18			U2OS			Non-Target U937
	AC	SS	OR	AC	SS	OR	AC
SO6	24.2	314.6	740.6	13.1	473.3	236.8	470.7
Tf-SO6	1.3	5.4	9.8	0.1	4.2	1.0	>100
Ocy	58.9	751.9	1219	73.9	593.5	196.7	1076
αEGFR1-Ocy	0.3	2.8	9.1	1.3	12.1	1.8	>100
αHer2-Ocy	8.8	6.8	28.8	3.7	11.1	0.7	>100

## Data Availability

The original contributions presented in this study are included in the article/Supplementary Material. Further inquiries can be directed to the corresponding authors.

## Supplementary Materials

The following supporting information can be downloaded at: https://www.mdpi.com/article/10.3390/toxins17060308/s1, Figure S1: Flow cytometry analysis of TfR1 expression; Figure S2: Flow cytometry analysis of EGFR1 expression; Figure S3: Flow cytometry analysis of Her2 expression; Figure S4: Flow cytometry analysis of TfR1, EGFR1 and Her2 expression levels in U937 non-target cells; Figure S5 (A, B, C, D): Development of U937 as 3D models and Its’ dose–response viability curves; Figure S6: Complete dose–response curves of RIPs; Figure S7 (A, B, C, D): Time-dependent caspase 3/7 activation and viability in RD18 and U2OS 2D and 3D models.

## Abbreviations

The following abbreviations are used in this manuscript:

AC	Adherent cells
IC_50_	Concentration inhibiting 50% of cell viability
ITs	Immunotoxins
mAb	Monoclonal antibody
Ocy	Ocymoidine
OR	Organoids
RIPs	Ribosome-inactivating proteins
SO6	Saporin
SS	Single spheroids
Tf	Transferrin
