# Supplementary material for: Comparative Efficacy of Ribosome-Inactivating Protein-Containing Immunotoxins in 2D and 3D Models of Sarcoma
Source: Toxins (Basel). 2025 Jun 18;17(6):308. doi: 10.3390/toxins17060308 (PMC12197652; doi:10.3390/toxins17060308)
Supplement: Supplementary file 1 [file toxins-17-00308-s001.zip › toxins-3656371-supplementary.pdf]

Supplementary Materials: Comparative Efficacy of Ribosome-Inactivating Protein-Containing Immunotoxins in 2D and 3D Models of Sarcoma

Giulia Calafato, Massimo Bortolotti, Letizia Polito and Andrea Bolognesi

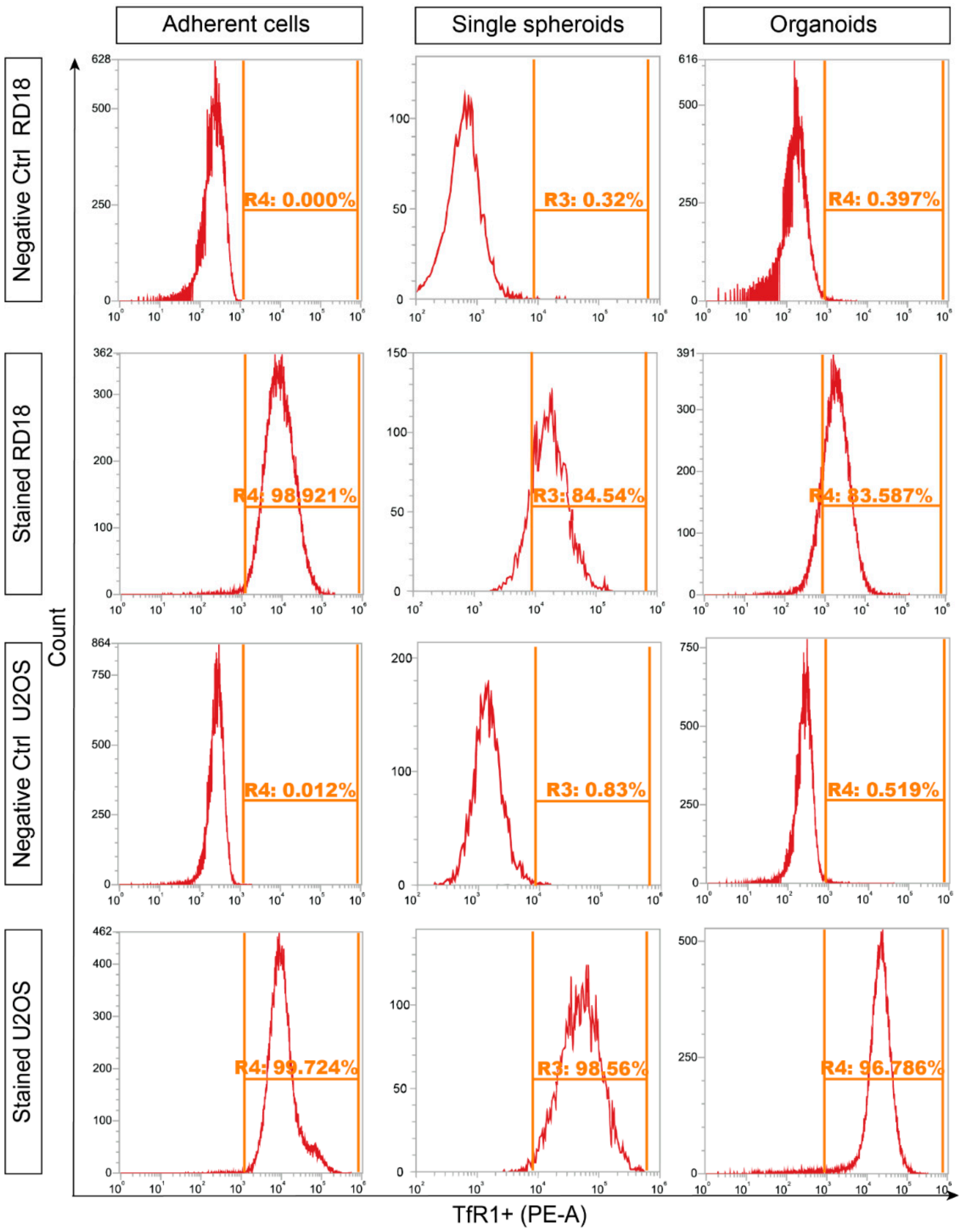

Figure S1. Flow cytometry analysis of TfR1expression. Flow cytometry graphs of RD18 and U2OS adherent cells (AC), single spheroids (SS) and organoids (OR). Stained samples were incubated with antiTfR1-PE (1:50) while negative control with PBS-containing 1% FBS. Graphs are representative of two independent experiments, each conducted in triplicate. AC and OR were analyzed by flow cytometry Attune NxT cell analyzer (UCLA) while SS by flow cytometry Cytoflex analyzer (CRBA).

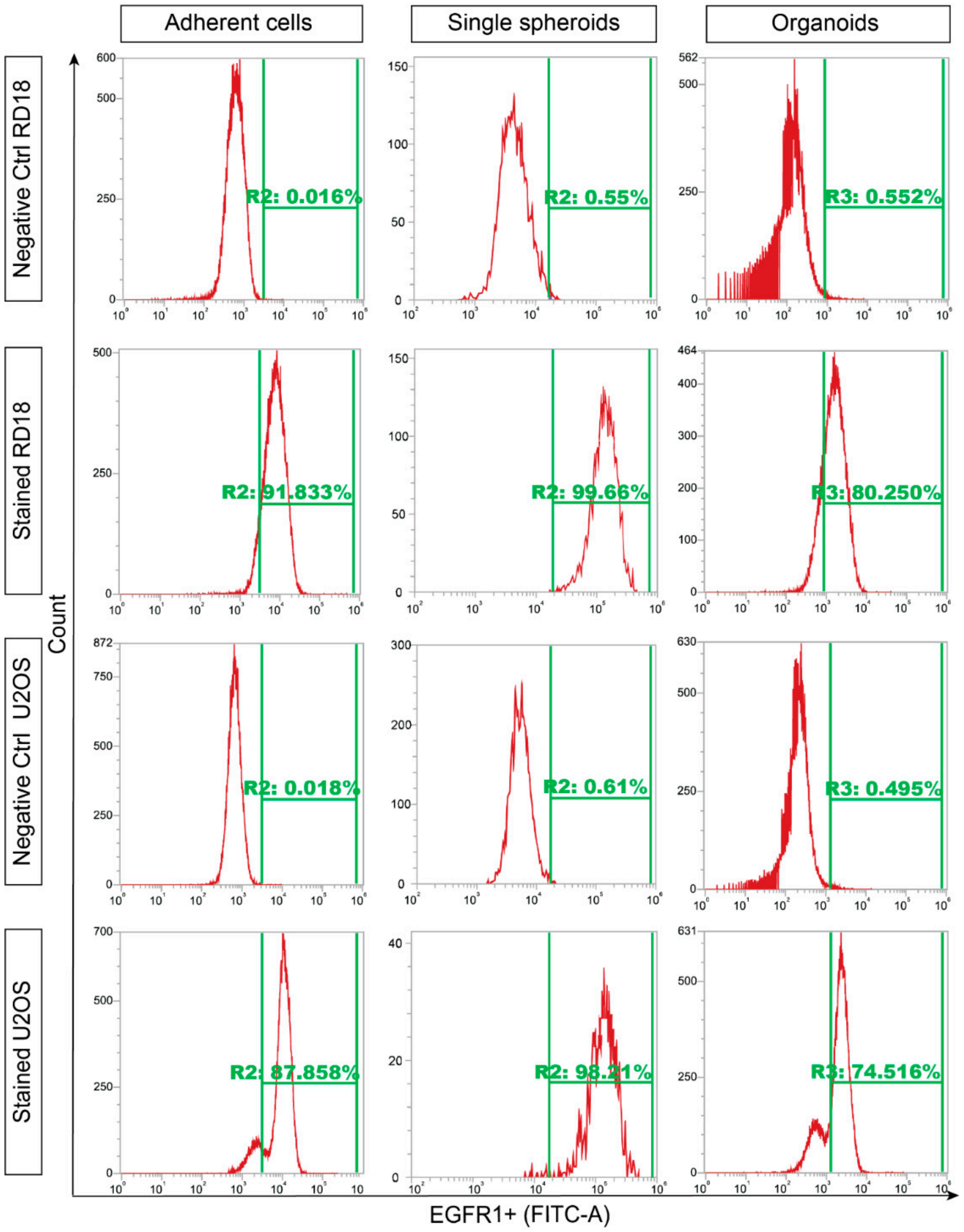

Figure S2. Flow cytometry analysis of EGFR1 expression. Flow cytometry graphs of RD18 and U2OS adherent cells (AC), single spheroids (SS) and organoids (OR). Stained samples were incubated with antiEGFR1-FITC (1:50) while negative control with PBS-containing 1% FBS. Graphs are representative of two independent experiments, each conducted in triplicate. AC and OR were analyzed by flow cytometry Attune NxT cell analyzer (UCLA) while SS by flow cytometry Cytoflex analyzer (CRBA).

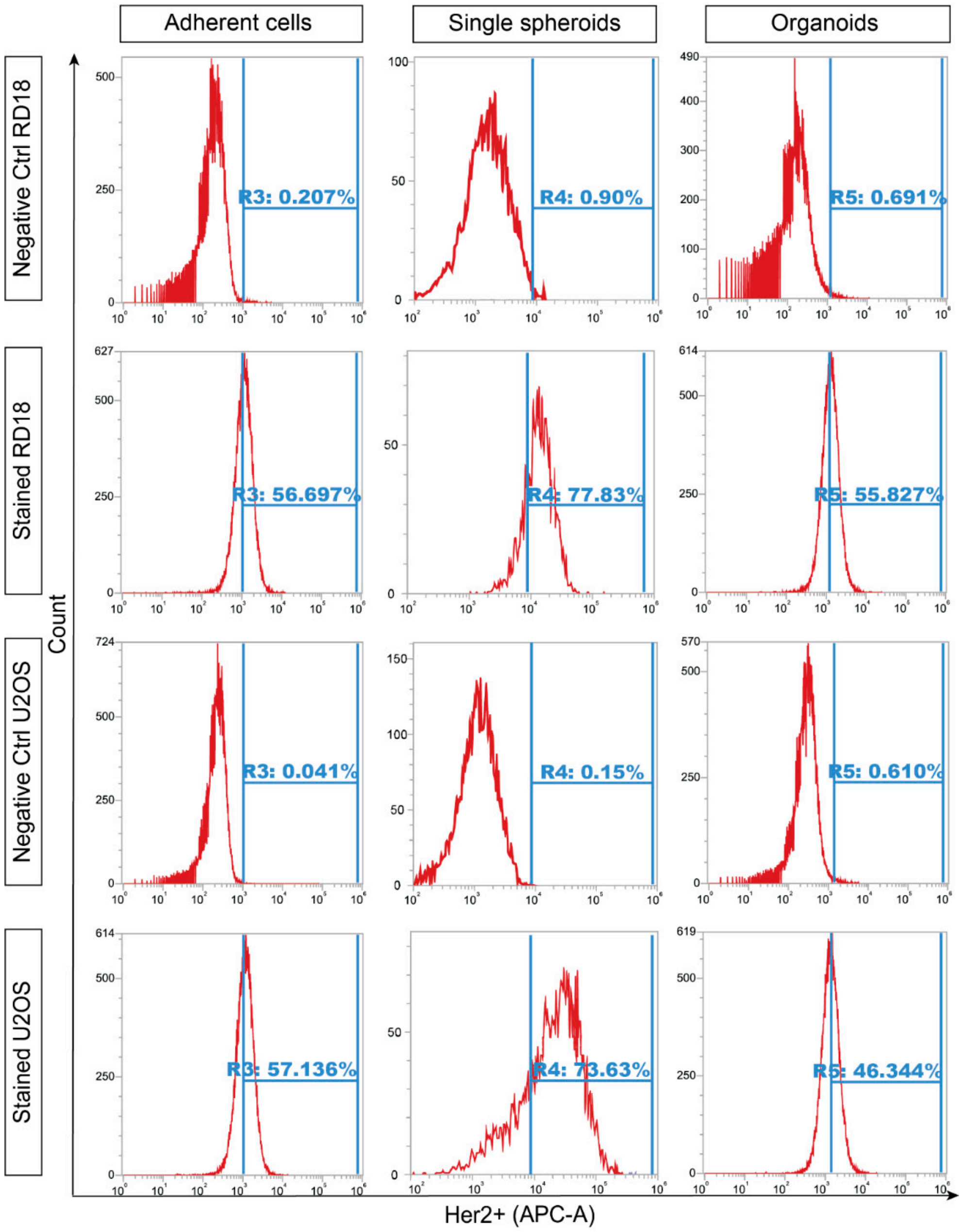

Figure S3. Flow cytometry analysis of Her2 expression. Flow cytometry graphs of RD18 and U2OS adherent cells (AC), single spheroids (SS) and organoids (OR). Stained samples were incubated with antiHer2-APC (1:50) while negative control with PBS-containing 1% FBS. Graphs are representative of two independent experiments, each conducted in triplicate. AC and OR were analyzed by flow cytometry Attune NxT cell analyzer (UCLA) while SS by flow cytometry Cytoflex analyzer (CRBA).

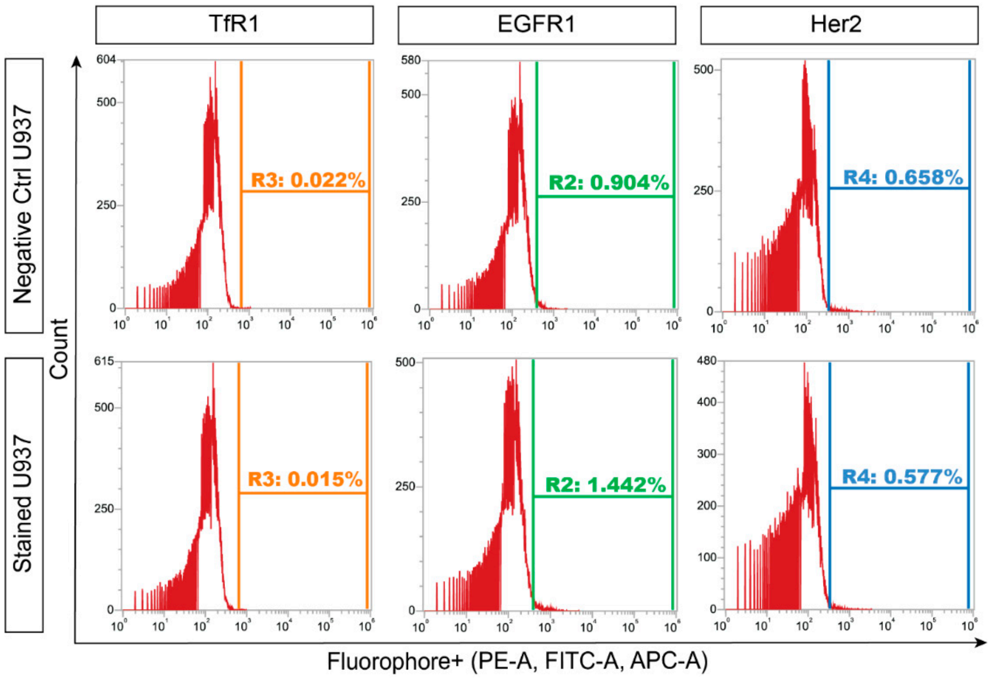

Figure S4. Flow cytometry analysis of TfR1, EGFR1 and Her2 expression in U937 non target cells. Stained samples were incubated with antiTfR1-PE (1:50), antiEGFR1-FITC (1:10) and antiHer2-APC (1:10) while negative controls were incubated with PBS-containing 1% FBS. Graphs are representative of two independent experiments, each conducted in triplicate. Cells were analyzed by flow cytometry Attune NxT cell analyzer (UCLA).

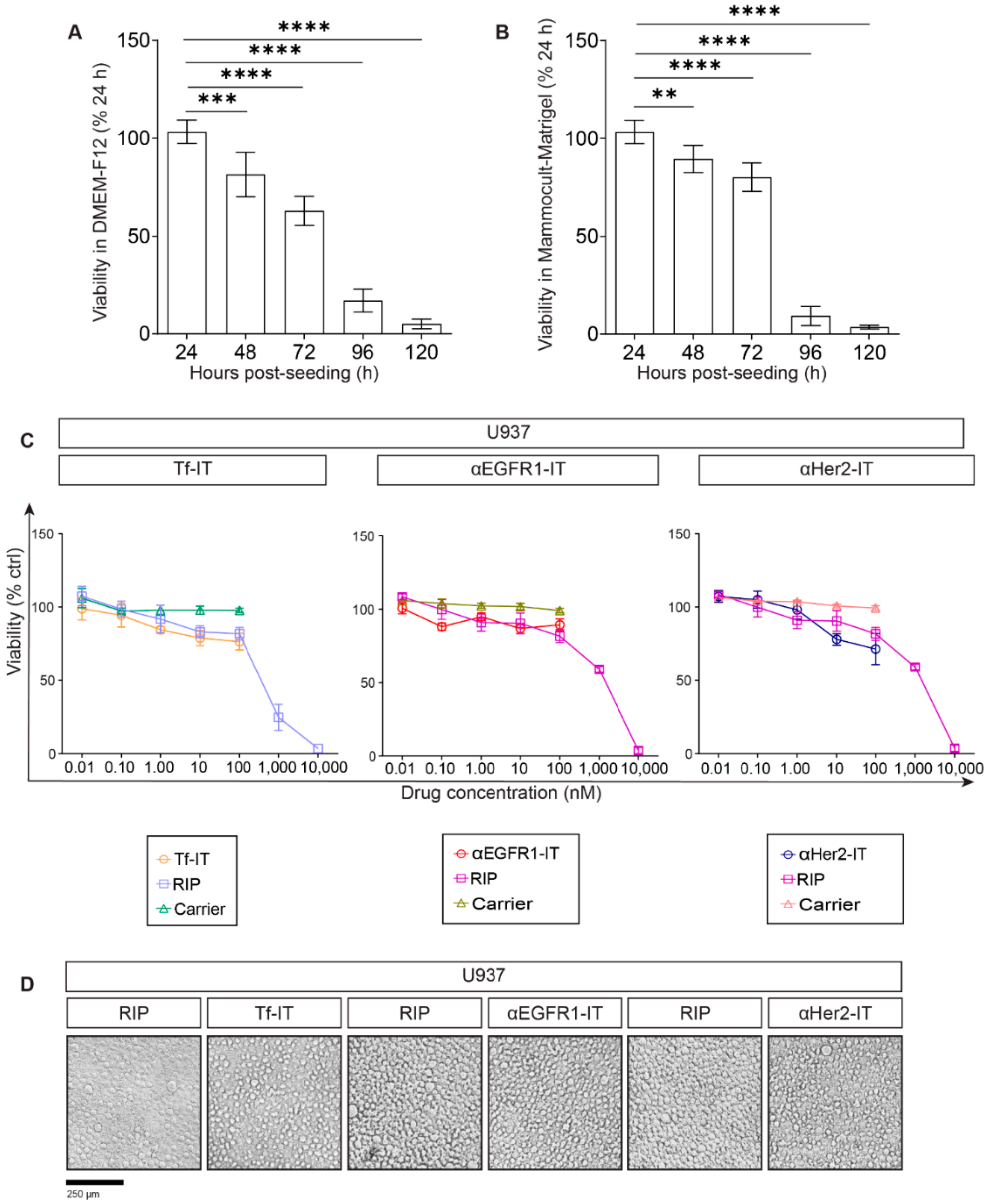

Figure S5. Development of U937 as 3D models and ITs dose-response viability curves. **A.** Time-course viability of U937 cells grown in DMEM-F12 complete medium. Viability was evaluated using the 3D luminometric ATP assay every 24 h. Data have been normalized to viability at 24 h post-seeding and reported as mean ± SD (Prism v8). **B.** Time-course viability of U937 cells (cells-matrigel mixture) in Mammocult complete medium. Viability was evaluated using the luminometric ATP assay every 24 h. Data have been normalized to viability at 24 h post-seeding and reported as mean ± SD (Prism v8). **C)** Dose-response curves in U937 non-target cells. U937 were treated for 72 h with IT or unconjugated RIP or carrier with the indicate scalar dilutions. Viability was evaluated using MTS reduction-based assay. For each condition, viability was expressed as the percentage of mean value, relative to the mean of the untreated controls. Results are shown as the mean (percentage) ± SD (Prism v8). IC_{50} was calculated using non-linear regression (Prism v8). **D)** Representative imaging of viability assay in U937 non-target cells treated with the indicated IT or RIP for 72 h at 100 nM. Imaging was performed using a phase-contrast microscope with a digital camera from Nikon Eclipse TS100 (100× magnification); scale bar=250 µm. Experiments were independently repeated two times, each in triplicate, and values are reported as mean ± SD. Data were analyzed with One-way ANOVA followed by Tukey's post-hoc test. * $P < 0.05$, ** $P < 0.01$, *** $P < 0.001$, **** $P < 0.0001$ (Prism v8).

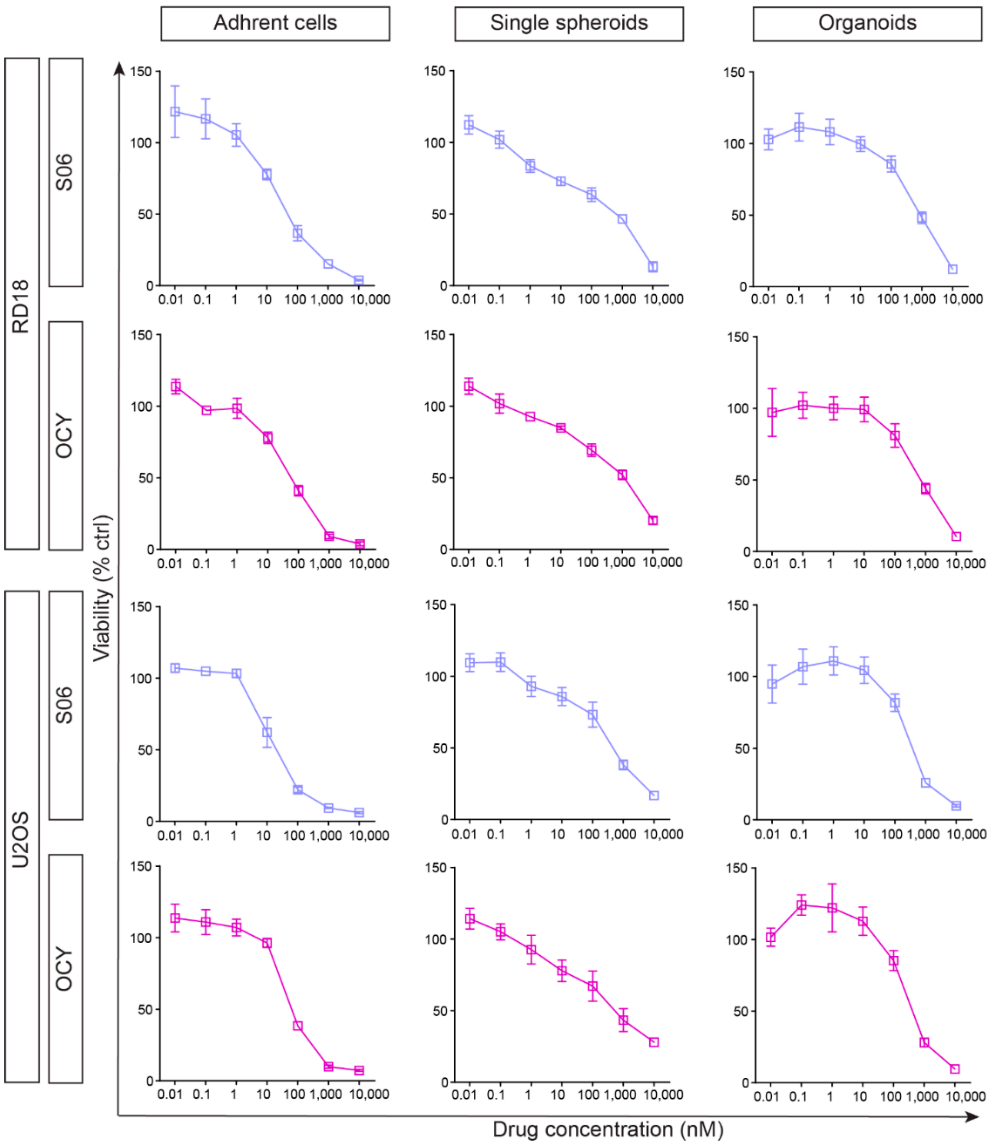

Figure S6. Complete dose-response curves of RIPs. Dose-response curves in RD18 and U2OS-derived adherent cells (AC), single spheroids (SS) and organoids (OR) treated for 72 h with the indicated scalar concentrations of unconjugated RIP (SO6 and Ocy). Viability was evaluated using MTS reduction based- (AC,) and 3D ATP-assay (SS and OR). Experiments were independently repeated two (AC and OR) and four (SS) times, each in triplicate. IT, RIP or carrier viability values were expressed as percentage of controls (PBS) and reported as mean ± SD (Prism v8).

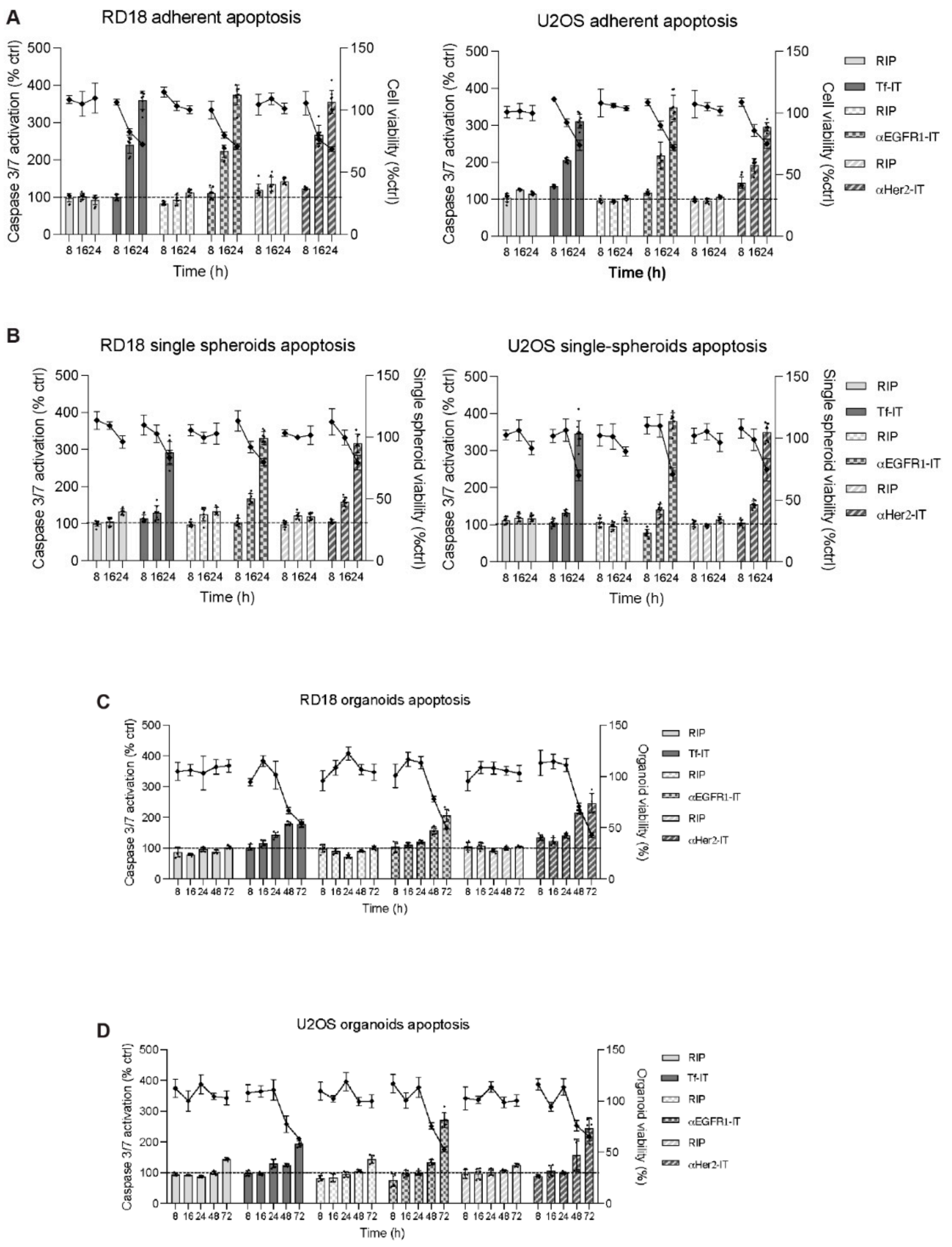

Figure S7. Time-dependent caspase 3/7 activation and viability in RD18 and U2OS 2D and 3D models. **A)** Caspase 3/7 activation (Y left axis – bars) and corresponding viability (Y right axis – lines) in RD18 and U2OS adherent cells (AC). **B)** Caspase 3/7 activation (Y left axis – bars) and corresponding viability (Y right axis – lines) in RD18 and U2OS single spheroids (SS). **C-D)** Caspase 3/7 activation (Y left axis - bars) and corresponding viability (Y right axis – lines) in RD18 and U2OS organoids (OR). RD18 and U2OS AC, SS and OR were exposed to IC_{50} of Tf-IT, αEGFR1-IT and αHer2-IT or corresponding RIP. Experiments were independently repeated two (AC and OR) or four (SS) times, each in triplicate. For each condition, caspase 3/7 activation value was normalized to the corresponding viability value and expressed as percentage of untreated controls (PBS). Data are reported as mean ± SD.
